# Membrane-Based Decolourisation and Purification of Starch Hydrolysates: A Systematic UF–NF Screening Study

**DOI:** 10.3390/membranes16070251

**Published:** 2026-07-22

**Authors:** Camila Cabeza, Amal El Gohary Ahmed, Michael Harasek

**Affiliations:** 1 Institute of Chemical, Environmental & Bioscience Engineering E166, Technische Universität Wien, 1060 Vienna, Austriamichael.harasek@tuwien.ac.at (M.H.); 2 Competence Center CHASE GmbH, Ghegastraße 3 Top 3.2, 1030 Vienna, Austria

**Keywords:** ultrafiltration (UF), nanofiltration (NF), starch hydrolysates, decolourisation, membrane screening, polymeric membranes, fouling behaviour, downstream processing

## Abstract

Membrane-based processes offer promising sustainable alternatives for the decolourisation and purification of starch hydrolysates, yet membrane selection and operating conditions remain the most critical challenges. This study systematically evaluates the performance of polymeric ultrafiltration (UF) and nanofiltration (NF) membranes for starch hydrolysate syrup treatment. Experiments were conducted in a lab-scale cross-flow filtration system using five UF and three NF flat-sheet polymeric membranes under varying temperatures, transmembrane pressures, and feed concentrations. Separation performance was assessed through colour removal, sugar recovery, permeate flux, and alongside indicators of fouling behaviour. UF membranes with molecular weight cut-offs of 100, 70, and 5 kDa exhibited the most favourable performance at 60 °C and 8 bar, achieving partial colour removal (18–32%) with high permeate fluxes (84–130 kg·m^−2^·h^−1^) and limited sugar losses (0.7–19.9%). NF membranes showed significantly higher colour rejection (32–100%) but were associated with substantial sugar losses (up to 96%), limiting their applicability for selective decolourisation; however, their high sugar retention capacity suggests potential for product concentration and the removal of low-molecular-weight impurities. Overall, UF represents a suitable approach for partial colour removal in starch hydrolysates, while NF may be better suited for product concentration and the removal of low-molecular-weight impurities, as well as auxiliary applications such as water recovery. These findings provide a systematic basis for membrane selection and process optimisation in industrial starch hydrolysate purification.

## 1. Introduction

The production of starch hydrolysates is a key process in the food and biochemical industries, generating syrups widely used as sweeteners, stabilising agents, and fermentation substrates. However, these streams are complex mixtures containing not only sugars but also residual proteins, salts, colloidal matter, and coloured compounds formed during hydrolysis, particularly melanoidins, coloured molecules produced via Maillard reactions [1,2]. The presence of these impurities adversely affects product quality, stability, and downstream processing, making efficient purification and decolourisation essential.

Conventional treatment methods, including ion exchange, activated carbon adsorption, and chemical clarification using calcium oxide and carbon dioxide, are widely applied at an industrial scale. However, these approaches are often associated with long processing times, high chemical and energy consumption, and significant environmental burdens [3,4,5]. Moreover, their limited selectivity and efficiency, typically removing only a fraction of non-sugar compounds, have motivated the search for more sustainable and efficient alternatives.

Membrane technology has emerged as a promising, industrially available solution due to its mild operating conditions, modularity, and ability to separate components based on molecular size and physicochemical properties. In particular, ultrafiltration (UF) and nanofiltration (NF) have demonstrated strong potential for the purification of carbohydrate-rich solutions, enabling improved product quality while reducing waste generation and resource consumption [6,7]. UF is generally effective for removing macromolecules and colloidal impurities, whereas NF can target smaller solutes and colour compounds, offering complementary separation capabilities. Nevertheless, the performance of these processes is highly dependent on membrane characteristics and operating conditions, and trade-offs between colour removal, sugar retention, and fouling behaviour remain a key challenge [8,9,10,11,12].

Previous studies have demonstrated the potential of membrane processes for the purification and decolourisation of carbohydrate-rich streams, including sugar beet syrups, cane sugar solutions, molasses, starch hydrolysates, and oligosaccharide mixtures. In sugar processing, UF and NF have been successfully applied for the removal of colour-forming compounds, proteins, colloids, and inorganic impurities, with reported colour reductions ranging from approximately 10% to more than 80%, depending on membrane MWCO and operating conditions [11,12,13,14,15,16,17]. Several authors have shown that membrane pore size strongly influences the balance between colour removal and sugar recovery, with tighter UF and NF membranes generally achieving higher colour rejection but often at the expense of increased sugar retention and reduced selectivity [7,11,15,18].

Similar observations have been reported for oligosaccharide and carbohydrate fractionation processes. Membranes with MWCO values between 500 and 2000 Da have been investigated for the separation of sucrose, fructo-oligosaccharides, and other carbohydrate fractions, demonstrating that membrane selectivity is strongly dependent on molecular size distribution, operating conditions, and solute–membrane interactions [7,15,18,19]. While these studies provide valuable insights into membrane separation mechanisms, they primarily focus on specific membranes, model solutions, or individual operating conditions.

In the case of starch hydrolysates, only a limited number of studies have systematically evaluated membrane performance under realistic industrial conditions. Previous investigations have demonstrated that UF membranes with MWCO values between 5 and 100 kDa can achieve partial colour removal while maintaining high sugar recovery, whereas NF membranes may provide additional concentration and low-molecular-weight impurity removal [8,20,21,22]. However, comparative screening studies that simultaneously evaluate multiple commercial membranes across different MWCO ranges and operating conditions remain scarce.

Despite extensive research, there is still a lack of systematic approaches to membrane selection under realistic industrial conditions. In particular, comparative screening studies that simultaneously evaluate multiple membranes and process parameters with real products remain limited, hindering the identification of optimal configurations for starch hydrolysate purification products.

In this context, the present work aims to systematically assess the performance of polymeric UF and NF membranes for the decolourisation and purification of starch hydrolysates. A comprehensive screening methodology was implemented, evaluating multiple membranes under varying temperatures, transmembrane pressures, and feed concentrations. The study focuses on key performance indicators, including permeate flux, colour removal, solute rejection, fouling behaviour, and sugar recovery, to identify optimal membrane–process combinations. The results demonstrate that UF membranes provide an effective balance between colour removal and sugar recovery, while NF, although limited for selective decolourisation, shows potential for complementary applications such as concentration, impurity removal and water recovery. These findings contribute to a more rational basis for membrane selection and process design in industrial starch hydrolysate purification.

## 2. Materials and Methods

### 2.1. Starch Hydrolysate Solution

This study used a hydrolysate syrup with a dextrose equivalent (DE) of 40, obtained through enzymatic hydrolysis using commercial amylolytic enzymes. The syrup, derived primarily from maize and wheat, was supplied by AGRANA Stärke GmbH (Pischelsdorf, Austria), an Austrian starch processing company. The degree of hydrolysis, expressed as the DE value, determines the composition and functional properties of the starch hydrolysate solution [23]. The DE40 syrup comprises a balanced mixture of glucose, maltose, and maltodextrins, with a high concentration of short-chain carbohydrates suitable for various industrial applications, including food and beverage production, fermentation processes, pharmaceuticals, and bio-based materials [24,25,26] (see Table 1).

Prior to experiments, the syrup was diluted with deionised water to obtain working concentrations of 20–30% dry solids (DS) for UF and 15–20% DS for NF. When required, pH was adjusted to 4.5 ± 0.5 using dilute NaOH or H_2_SO_4_ (0.5% *w*/*v*), representing typical industrial conditions and ensuring stability of colour compounds and membrane performance [21,27]. The feed concentrations were selected based on membrane type and MWCOs to be studied, considering size exclusion and selective permeability, key mechanisms governing solute retention and separation efficiency.

### 2.2. Experimental Setup and Membranes

**Figure 1:**
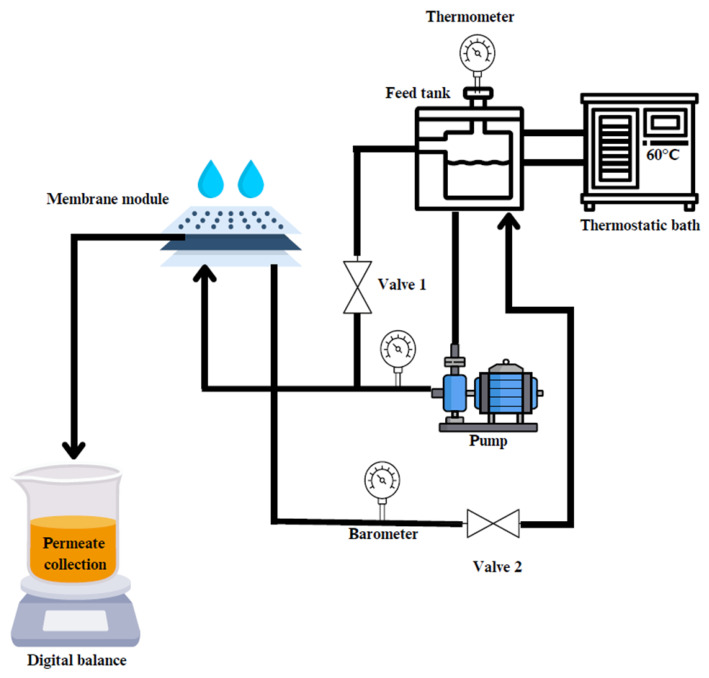
Lab-scale cross-flow filtration unit (Model OS-MC-01). Effective membrane area = 0.008 m^2^ (0.04 m × 0.2 m). Reprinted from [28].

Trials were conducted using a lab-scale cross-flow filtration unit (OS-MC-01 model, OSMO Membrane Systems GmbH, Leonberg, Germany) at TU Wien. The unit system comprised a flat-sheet membrane module (effective area: 0.008 m^2^), a 2 L temperature-controlled feed tank, and a high-pressure piston pump (CAT model 231) with a flow capacity of 3.7 L min^−1^ and a maximum applied pressure of 64 bar. Experiments were performed in a short-batch mode with continuous retentate recirculation and permeate collection until ~70% volume recovery. Figure 1 illustrates the schematic flow diagram of the experiment setup.

A total of eight flat-sheet polymeric membranes (five UF and three NF) with different molecular weight cut-offs (MWCO) and polymeric materials were selected to cover a broad separation range relevant to starch hydrolysate purification and decolourisation. The membrane set included loose and tight UF membranes as well as NF membranes, allowing the systematic evaluation of size-exclusion effects, membrane selectivity, and fouling behaviour across different separation regimes. The selected membranes were chosen based on their commercial availability, suitability for food processing applications, thermal stability under the investigated operating conditions, and their potential applicability for industrial starch hydrolysate treatment.

Prior to each experiment, membranes were pre-hydrated in deionised water for 20 min and subsequently compacted under corresponding operating conditions for an additional 20 min to minimise the influence of membrane conditioning effects and ensure stable baseline performance. The initial pure water flux (PWF) was then measured and used as a reference for subsequent fouling assessment. Following starch hydrolysate filtration, the feed solution was discharged, and the membrane was gently rinsed with demineralised water to remove residual feed from the system without applying any dedicated cleaning procedure. Subsequently, a second pure water flux measurement was performed under the same operating conditions. The difference between the initial and final PWF values was used as an indicator of membrane fouling. To avoid any interference from previous filtration runs and to ensure experimental reproducibility, a new membrane sample was used for each experiment. Detailed membrane characteristics, including membrane material, MWCO, manufacturer specifications, and operating limits, are summarised in Table 2.

### 2.3. Operating Conditions and Screening Procedure

A systematic screening approach was applied to evaluate membrane performance under controlled conditions. Experiments were conducted at temperatures between 40 and 60 °C. Transmembrane pressure ranged from 2 to 8 bar for UF, and different feed concentrations and up to 30 bar for NF. Cross-flow velocity was maintained at approximately 3.6 L·min^−1^ to minimise concentration polarisation. Table 3 presents the specific operating conditions for each membrane type.

At the start of each experiment, approximately 2 L of prepared feed solution was introduced into the system and equilibrated for 15 min. Baseline measurements (pH, conductivity, °Brix, and colour) were recorded. Filtration was performed with continuous retentate recirculation, and the permeate was collected gravimetrically. Experiments were terminated at ~70% recovery to ensure comparability across membranes. Between experiments, the system was rinsed with deionised water. Pure water flux (PWF) was measured before and after each run to assess membrane performance and fouling.

### 2.4. Analytical Method

Process performance was evaluated using standard analytical techniques. Colour was determined spectrophotometrically at 420 nm using a UV/Vis spectrophotometer (Shimadzu UV-1800, Shimadzu Corporation, Kyoto, Japan) following ICUMSA methodology, with results expressed in ICUMSA Units (IU) [29].

Total dissolved solids were measured as °Brix using a digital refractometer (KRÜSS DR6200-T, A. KRÜSS Optronic GmbH, Hamburg, Germany) [30]. Carbohydrate composition (glucose, maltose, maltotriose, and higher oligosaccharides) was analysed by high-performance liquid chromatography (HPLC) with refractive index detection. Additional size exclusion chromatography (SEC) analyses were performed to assess molecular weight distribution.

To ensure process stability, pH and conductivity levels in both the permeate and feed were continuously monitored using a multiparameter (VWR pHenomenal^®^ MU 6100 H, VWR International, Vienna, Austria). All these critical parameters were measured every 20 min, with specific analyses conducted as needed throughout the trials, ensuring the accuracy and reliability of the collected data.

### 2.5. Performance Evaluation

Membrane performance was assessed using standard indicators. Solute rejection (R) was calculated as the relative reduction in solute concentration between feed and permeate. Where R is the Rejection coefficient (%), representing the membrane’s ability to retain a given solute; $C_{p}$ is the solute concentration in the permeate (solution that has passed through the membrane); Cf is the solute concentration in the feed (solution before filtration).

(1) $$R=\left(1-\frac{C_{p}}{Cf}\right)*100\%$$

Permeate flux (J) was determined as the permeate mass per membrane area and time, where $m_{p}$ is the permeate mass, A is the effective membrane area, and t is the collection time. Flux data provided insight into hydraulic performance and the influence of operating conditions.

(2) $$J=\frac{m_{p}}{A*t}$$

Pure water flux (PWF), representing the baseline water-permeability performance of the membrane, was established by measuring the flux of deionised water under defined operating conditions. PWF was recorded both before and after filtration runs to serve as a reference for fouling and cleaning assessments. Where PWF (kg/m^2^·h) is calculated according to Equation (3), indicating the rate at which deionised water permeates through the membrane; m (kg) is the mass of permeate water collected during the test; A (m^2^) is the effective membrane area; t (h) is the time of collection.

(3) $$PWF=\frac{m}{A\;*\;t}$$

### 2.6. Membrane Selection and Semi-Quantitative Performance Assessment

To facilitate the comparison of membrane performance and support the final membrane selection, a semi-quantitative key performance indicator (KPI) approach was applied. The evaluated performance criteria included permeate flux, colour removal, sugar loss, further purification potential (salt and low-molecular-weight impurity removal), and flux reduction as an indicator of fouling propensity. For each criterion, membrane performance was classified into qualitative categories (Very Low, Low, Moderate, High, and Very High) based on the relative performance observed during the screening experiments. These categories were subsequently converted into numerical scores ranging from 1 to 5, where higher scores represented more favourable performance. The individual scores were summed to obtain an overall membrane performance score, which was used as a supporting tool for membrane selection. The final membrane choice was based not only on the total score but also on practical considerations, including membrane availability, suitability for food applications, preservation of the DE40 carbohydrate profile, and potential for industrial implementation.

### 2.7. Statistical Analysis

Statistical analysis was performed using one-way analysis of variance (ANOVA) to evaluate differences between membranes and operating conditions. A significance level of *p* < 0.05 (95% confidence interval) was applied. Calculated parameters, including rejection, flux, and fouling indicators, were treated as dependent variables. All statistical analyses were performed in Microsoft Excel.

## 3. Results and Discussion

### 3.1. Ultrafiltration Screening

**Figure 2:**
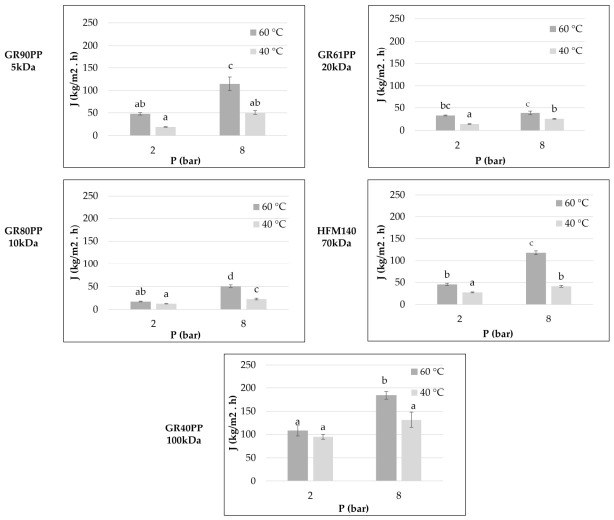
Influence of operating conditions (temperature and pressure) on total permeate fluxes during UF processes. Different lowercase letters indicate groups that differ significantly (*p* < 0.05) based on Tukey’s HSD post hoc test; bars sharing the same letter are not significantly different.

The ultrafiltration (UF) screening systematically evaluated five flat-sheet UF membranes under two temperatures (40 and 60 °C) and two transmembrane pressures (2 and 8 bar) to compare their permeate fluxes, rejection performance, and fouling. The resulting trends in total permeate flux are summarised in Figure 2.

As expected for a pressure-driven membrane process, increasing transmembrane pressure (TMP) generally enhanced permeate flux by increasing the hydraulic driving force across the membrane. At low TMP, the flux increase was approximately proportional to pressure, indicating that hydraulic resistance dominated the transport behaviour. At higher TMP, however, the increase became less pronounced, suggesting the onset of concentration polarisation and fouling, which introduce additional resistance and progressively limit further flux enhancement. Similar behaviour has been described for UF systems treating sugar-rich streams, where high pressure may improve productivity but can also intensify fouling and increase operating costs if applied beyond the optimal range [1,8,31]. While Hamachi et al. [11] likewise reported that permeate flux increases with TMP and cross-flow velocity, it decreases with decreasing pore size.

Temperature also had a pronounced effect on UF performance. Raising the temperature from 40 to 60 °C consistently increased permeate flux, mainly due to reduced feed viscosity and improved mass transfer. This effect was particularly evident for the tighter membranes, where viscosity-related resistance constitutes a larger fraction of the total resistance. Similar temperature-dependent flux enhancement has been reported previously for carbohydrate-rich solutions [7,11,32]. In practical terms, operating at lower temperatures would require substantially larger membrane areas to achieve the same throughput, which is generally unfavourable for industrial application [8,33].

Marked differences among the membranes were observed depending on molecular weight cut-off (MWCO) and membrane material. The looser membranes, HFM140 (70 kDa) and GR40PP (100 kDa), exhibited the highest permeate fluxes, ranging from 118 to 184 kg·m^−2^·h^−1^, consistent with their lower intrinsic transport resistance. In contrast, the tighter membranes (20, 10, and 5 kDa) showed lower fluxes overall. However, the flux trend did not strictly follow nominal MWCO, indicating that membrane chemistry and fouling propensity also played an important role. The 5 and 10 kDa membranes, both based on polyethersulfone (PES), outperformed the 20 kDa polysulfone (PSU) membrane despite their lower nominal MWCO. This behaviour may be attributed to the more hydrophilic character commonly associated with PES membranes, which improves wetting and reduces adsorption fouling relative to PSU [34,35]. By contrast, the PVDF-based HFM140 membrane exhibited distinct behaviour that likely reflected stronger hydrophobic interactions and different boundary-layer formation tendencies, as has been reported for PVDF membranes in aqueous filtration systems [36].

**Figure 3:**
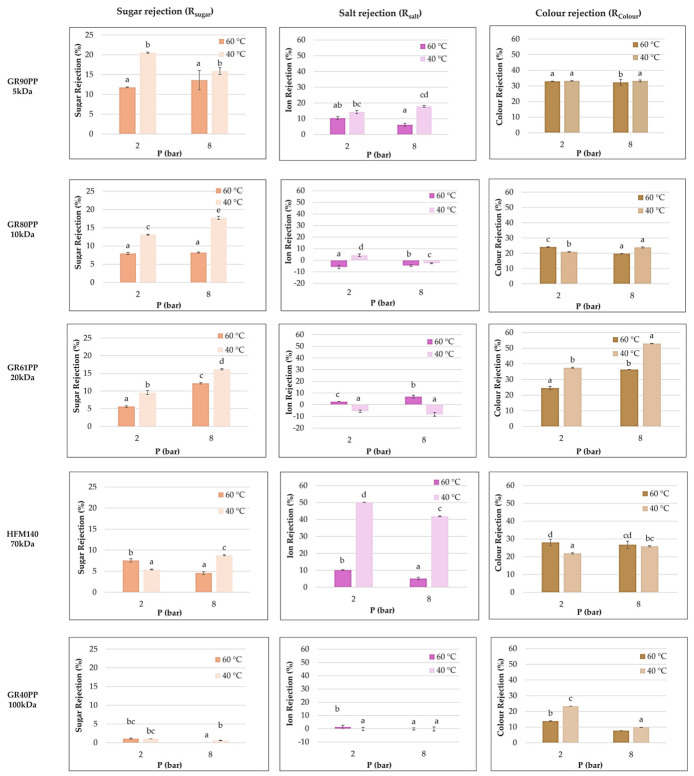
Influence of operating conditions (temperature and pressure) on specific rejection coefficients (Sugar, Salt, and Colour) during UF processes. Different lowercase letters indicate groups that differ significantly (*p* < 0.05) based on Tukey’s HSD post hoc test; bars sharing the same letter are not significantly different.

The corresponding rejection behaviour for sugars, salts, and colour compounds is shown in Figure 3. In this study, decolourisation refers to the rejection of coloured compounds, mainly Maillard-derived melanoidins, while sugar permeability refers to the transport of reducing sugars into the permeate, which constitutes the desired product stream in the UF stage. The overall objective of the UF screening was therefore to identify membranes and conditions that maximise sugar passage while retaining a meaningful fraction of colour-causing impurities.

Temperature had a measurable effect on solute rejection across the tested membranes. Increasing temperature generally reduced sugar rejection, which is consistent with lower feed viscosity, faster solute diffusion, and increased molecular mobility at elevated temperatures [37]. Tsuru et al. [38] additionally reported that the hydration layer at the pore wall may decrease with increasing temperature, effectively enlarging pore size and further facilitating solute passage. This effect was most evident for the tighter membranes, where steric hindrance and viscosity-related limitations are more pronounced. In contrast, the looser membranes showed only a limited temperature effect on sugar rejection because their pore structures already permitted substantial solute permeation. A similar trend has been reported for sugar-rich systems, where higher temperatures increase flux but often reduce retention of neutral solutes [7].

Colour rejection, however, was less consistently affected by temperature. Most membranes showed only minor changes in decolourisation performance between 40 and 60 °C, in agreement with previous observations that colour removal in UF is often influenced by the retention of high-molecular-weight colour compounds, particularly melanoidins, rather than by thermal effects [11]. However, the coloured fraction of starch hydrolysates is chemically complex and may also contain caramelization products, polyphenolic compounds, protein-sugar complexes, and other coloured macromolecular species. Therefore, decolourisation is likely governed by a combination of steric exclusion, adsorption onto the membrane surface or within membrane pores, hydrophobic interactions, electrostatic effects, and the retention of colloidal aggregates, rather than by pore-size effects alone. Nevertheless, some membranes, particularly the GR61PP 20 kDa membrane, achieved higher colour rejection at 40 °C, reaching a maximum of approximately 53%. This suggests that lower temperatures may favour retention of pigment aggregates by reducing their diffusivity and mobility, a behaviour also observed by [1]. Overall, the results indicate that while membrane pore size remains an important factor governing decolourisation, colour rejection in starch hydrolysate systems is likely controlled by multiple interacting physicochemical mechanisms rather than by size exclusion alone.

Pressure exerted a more limited and solute-dependent effect on rejection. In several cases, increasing TMP slightly increased sugar rejection, likely because higher convective solvent flow promoted steric hindrance under conditions of increased concentration polarisation. However, colour rejection remained relatively insensitive to pressure for most membranes, again indicating that colour retention was governed mainly by membrane pore structure rather than by hydrodynamic changes. Salt rejection was generally low or even negative, as expected for UF membranes, which are not designed to retain monovalent ions. An exception was the HFM140 70 kDa membrane, which exhibited an unexpectedly high salt rejection of approximately 50% at 40 °C. This behaviour may reflect membrane-specific charge effects or complex interactions between ions and the carbohydrate matrix. In concentrated sugar systems, electrostatic interactions, partial pore obstruction by large sugar molecules, competitive adsorption, viscosity-induced diffusion limitations, and osmotic effects can all contribute to atypical ion transport behaviour [7,39].

**Figure 4:**
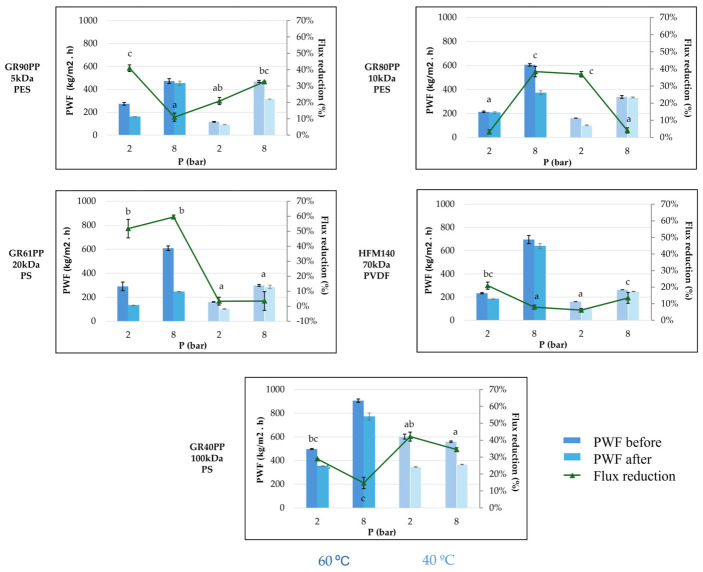
Influence of operating conditions (temperature and pressure) on membrane fouling and flux reduction during UF screening. Dark blue bars represent pure water flux before filtration (PWF before), light blue bars represent pure water flux after filtration (PWF after), and the green line indicates flux reduction (%). Results at 60 °C are shown on the left side of each graph, while results at 40 °C are shown on the right side. Different lowercase letters indicate groups that differ significantly (*p* < 0.05) based on Tukey’s HSD post hoc test; bars sharing the same letter are not significantly different.

The apparent fouling tendency, assessed through flux reduction measurements before and after filtration during UF screening, is presented in Figure 4. Overall, flux reduction tended to be lower at higher temperatures and pressures, suggesting that reduced viscosity and enhanced mass transfer may have contributed to lower foulant deposition and improved hydraulic performance during filtration. However, this trend was not universal. The GR80PP 10 kDa membrane and the GR61PP 20 kDa membrane exhibited the highest flux reductions, reaching 38% and 59%, respectively, under certain conditions. For the 10 kDa membrane, the lowest flux reduction was observed either at low pressure and high temperature or at high pressure and low temperature, indicating a strong interaction between hydrodynamic and thermal effects. By contrast, the 20 kDa membrane consistently performed better at 40 °C, suggesting that its apparent fouling tendency may have been influenced by temperature-dependent transport phenomena rather than pressure alone.

The HFM140 70 kDa, GR90PP 5 kDa, and GR40PP 100 kDa membranes showed lower flux reduction tendencies, especially at elevated temperature and pressure, with minimum flux reductions of 8%, 11%, and 15%, respectively. Higher temperatures likely improved foulant solubility and reduced residence time in the concentration-polarisation layer, while higher TMP enhanced permeation and reduced the opportunity for surface deposition [7]. It should be noted that these observations provide a qualitative indication of membrane fouling propensity under the tested conditions rather than a direct characterisation of fouling mechanisms.

Among all UF membranes, HFM140 showed the lowest flux decline overall, indicating the lowest apparent fouling propensity among the membranes evaluated. However, because this membrane is no longer commercially available, it was excluded from further evaluation despite its promising performance. Overall, the UF results show that membrane performance cannot be explained by MWCO alone; rather, permeability, rejection, and apparent fouling tendency were jointly determined by membrane chemistry, pore structure, operating conditions, and feed–membrane interactions.

### 3.2. Nanofiltration Screening

**Figure 5:**
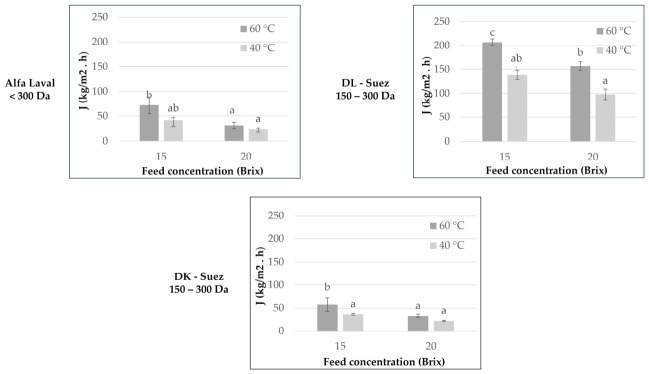
Influence of operating conditions (temperature and feed concentration) on total permeate fluxes during NF processes. Different lowercase letters indicate groups that differ significantly (*p* < 0.05) based on Tukey’s HSD post hoc test; bars sharing the same letter are not significantly different.

The nanofiltration (NF) screening aimed to identify membranes suitable for the final purification and concentration of starch hydrolysates. Three NF membranes were evaluated at 40 and 60 °C and at two feed concentrations (15 and 20 °Brix), while pressure was fixed at 30 bar. The resulting total permeate fluxes are shown in Figure 5.

Across all NF membranes, increasing temperature generally increased permeate flux. This behaviour is consistent with the reduction in feed viscosity and the increase in solvent mobility at elevated temperatures, both of which favour solvent transport through the denser active layer of NF membranes [38,40]. Unlike in UF, however, the magnitude of this flux increase was shaped more strongly by membrane structure, free-volume pathways, and charge-based selectivity. Solute fluxes increased much less than water flux, indicating that temperature influenced solvent transport more strongly than solute passage.

Feed concentration had a clear effect on water flux. Lower feed concentration (15 °Brix) consistently resulted in higher permeate fluxes, whereas increasing the concentration to 20 °Brix reduced water transport due to higher osmotic pressure and viscosity. This behaviour is typical for NF treating carbohydrate-rich streams, where the effective driving force is given by the difference between applied pressure and osmotic pressure (ΔP − Δπ) [41,42]. By contrast, sugar and salt fluxes changed only modestly with concentration, indicating that solute transport remained governed primarily by membrane selectivity rather than by osmotic effects. Because pressure was fixed at 30 bar, the observed differences can be attributed mainly to concentration- and temperature-related effects rather than TMP variation. This operating pressure was selected because it is sufficiently high to maintain practical fluxes in sugary feeds without introducing excessive membrane compaction [43].

The three NF membranes displayed clearly different behaviours. The DL-Suez membrane behaved as the loosest membrane, showing the highest sugar and salt fluxes and the lowest rejection. This is consistent with its higher nominal permeability and lower MgSO_4_ retention specified by the manufacturer. In contrast, DK-Suez and the Alfa Laval membrane exhibited tighter separation behaviour, with stronger retention of sugars, salts, and colour compounds, consistent with their denser thin-film composite structures and higher nominal ion rejection. These membrane-specific differences confirm that manufacturer specifications can explain the broad performance ranking, although the actual behaviour in starch hydrolysate systems is also influenced by complex feed–membrane interactions.

**Figure 6:**
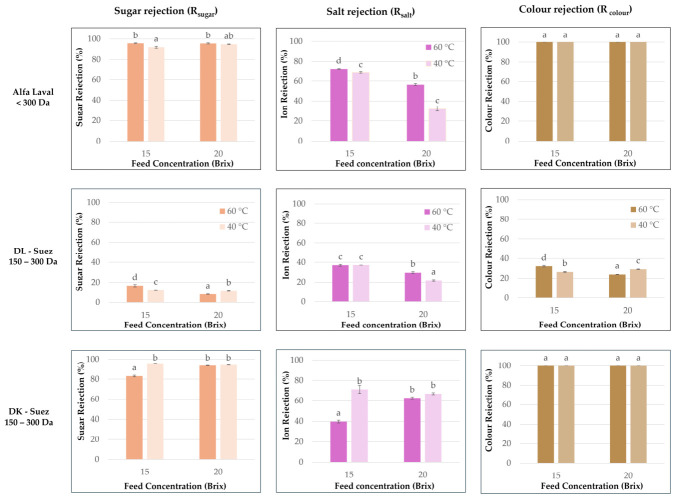
Influence of feed concentration and temperature on specific rejection coefficients (sugar, salt, and colour) for the NF membranes tested. Different lowercase letters indicate groups that differ significantly (*p* < 0.05) according to Tukey’s HSD post hoc test; bars sharing the same letter are not significantly different.

The rejection behaviour of the NF membranes is shown in Figure 6. In the NF stage, the retentate represents the desired product stream; consequently, the objective differed from that of the UF stage. Here, high sugar retention was desirable, while colour and low-molecular-weight impurity passage to the permeate could be advantageous depending on the intended process configuration.

Sugar rejection remained high for the tighter membranes and was only weakly affected by feed concentration. This is consistent with steric exclusion being the dominant mechanism governing the retention of neutral sugars in NF systems. Similar observations have been reported for sucrose- and reducing-sugar-containing solutions, where dilution has little effect on sugar retention [7,44]. In contrast, ion rejection was much more sensitive to feed concentration. For example, the Alfa Laval membrane showed a decline in ion rejection from an average of 70.5% at 15 °Brix to 44.3% at 20 °Brix, while DL-Suez decreased from 37.1% to 25.7%, respectively. This behaviour can be explained by increased osmotic pressure, higher viscosity, weaker back-diffusion, and charge shielding at higher sugar concentrations, all of which reduce effective Donnan exclusion and promote ion passage [44].

Temperature had relatively little effect on rejection for most NF membranes, although membrane-specific deviations were observed. DK-Suez showed the clearest sensitivity: at 15 °Brix and 60 °C, it exhibited its lowest sugar and ion rejections, 83.1% and 39.8%, respectively, whereas under the remaining conditions, sugar rejection remained around 94.1% and ion rejection around 66.8%. This behaviour is consistent with the tendency of elevated temperature to reduce solute retention by increasing diffusion and weakening electrostatic exclusion [38]. In contrast, the Alfa Laval membrane showed increased ion rejection at higher temperature and concentration, possibly because reduced concentration polarisation or thermally induced structural changes improved selectivity, as also suggested by Guo et al. [7]. These results indicate that the effect of temperature on NF rejection is membrane-specific and depends on the interplay among membrane structure, charge effects, and concentration polarisation.

Among the membranes, DL-Suez achieved the greatest colour permeation and therefore the most pronounced decolourisation effect in the permeate. However, this came at the expense of substantial sugar loss, indicating limited selectivity between colour-forming compounds and carbohydrates. Consequently, although DL-Suez may be suitable in processes where the permeate is the desired stream, it is less appropriate for the present application, where the retentate is intended as the purified product. By comparison, DK-Suez and the Alfa Laval membrane showed much better sugar retention and are therefore more suitable for downstream concentration and polishing. In particular, the selected Dk-Suez membrane achieved a sugar rejection of up to 95.4%, allowing effective concentration of the starch hydrolysate while maintaining the desired carbohydrate composition. This supports the interpretation that NF is not an efficient stand-alone decolourisation step for starch hydrolysates, as colour rejection was generally accompanied by similar sugar rejection behaviour. Instead, NF appears more appropriately suited as a complementary polishing and concentration step following upstream clarification or partial decolourisation by UF. Similar integrated concepts have been proposed for carbohydrate-rich streams such as cane molasses, where UF is combined with NF to reduce the load on downstream polishing steps [7,45].

The differences observed between the DK-Suez and DL-Suez membranes are consistent with previous studies on sugar and oligosaccharide separations. Although both membranes are classified as NF membranes, the DL membrane is generally considered a looser NF membrane, whereas the DK membrane exhibits tighter separation characteristics. Consequently, DK membranes typically provide higher carbohydrate retention and concentration efficiency, while DL membranes allow greater permeation of both sugars and low-molecular-weight compounds [7,18]. The higher sugar losses observed for DL-Suez in the present study therefore agree with its lower selectivity and confirm that, for starch hydrolysate purification where the retentate is the target product, DK-Suez represents the more suitable option.

**Figure 7:**
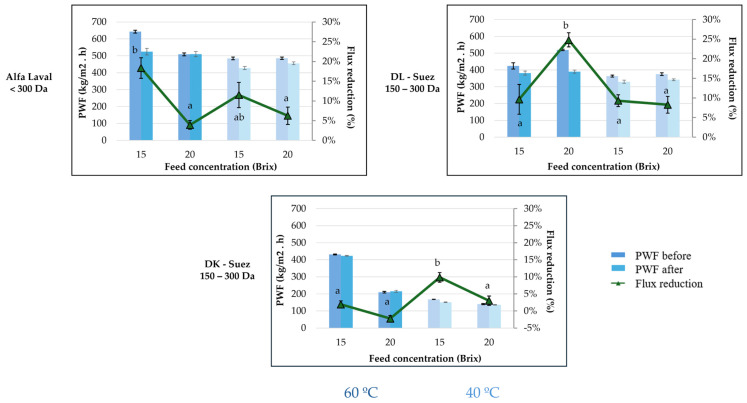
Influence of operating conditions (temperature and concentration) on membrane fouling and flux reduction during NF screening. Dark blue bars represent the pure water flux before filtration (PWF before), light blue bars represent the pure water flux after filtration (PWF after), and the green line indicates the flux reduction (%). Results obtained at 60 °C are shown on the left side of each graph, while results obtained at 40 °C are shown on the right side. Different lowercase letters indicate groups that differ significantly (*p* < 0.05) based on Tukey’s HSD post hoc test; bars sharing the same letter are not significantly different.

NF flux reduction behaviour and apparent fouling tendencies are summarised in Figure 7. Unlike in UF, no clear and consistent relationship was observed between operating conditions and flux reduction tendency. Instead, flux reduction depended more strongly on membrane type than on temperature or concentration alone. The highest flux decline was observed for DL-Suez, which reached 25% at 20 °Brix and 60 °C. The Alfa Laval membrane showed intermediate flux reduction behaviour, with a maximum reduction of 16%, whereas DK-Suez consistently exhibited the lowest flux decline, reaching only 2% under its best conditions. These results indicate that the observed differences in flux reduction may be associated with a combination of membrane tightness, surface chemistry, adsorption phenomena, concentration polarisation, and osmotic effects rather than with operating parameters alone. However, since fouling was assessed indirectly through changes in pure water flux before and after filtration, these observations should be interpreted as qualitative indicators of apparent fouling propensity rather than direct evidence of specific fouling mechanisms. This interpretation is consistent with the general view that fouling in pressure-driven membrane processes is multifactorial and strongly membrane-specific [46,47,48].

### 3.3. Selection of Optimal Membranes and Operating Conditions

The screening results showed that no single membrane could simultaneously maximise colour removal, minimise sugar loss, maintain high flux, and fully suppress fouling. Instead, membrane selection required balancing these competing criteria. To support this decision, the overall performance of all membranes was compared using a semi-quantitative KPI-based evaluation that considered flux, colour removal, sugar loss, further purification potential, and fouling propensity. The resulting comparison is summarised in Table 4.

Among the loose UF membranes, HFM140 (70 kDa) showed the best experimental performance, achieving approximately 27% colour removal, low sugar loss, and low flux decline at 60 °C and 8 bar. However, because this membrane is no longer commercially available, it was not considered suitable for further development. GR40PP (100 kDa) was therefore selected as the loose UF membrane. Although its colour removal was lower, it combined high flux, very low sugar loss, and acceptable fouling resistance, making it a robust pretreatment option.

Among the tighter UF membranes, GR90PP (5 kDa) was selected because it provided the best overall compromise between colour removal, sugar recovery, and fouling resistance. It achieved the highest colour removal among the selected candidates, approximately 32.3%, together with relatively low flux decline and stable operation under elevated temperature and pressure. Its PES-based active layer is also advantageous from a practical perspective because it is food-grade and resistant to hydrolytic degradation under warm aqueous conditions.

**Figure 8:**
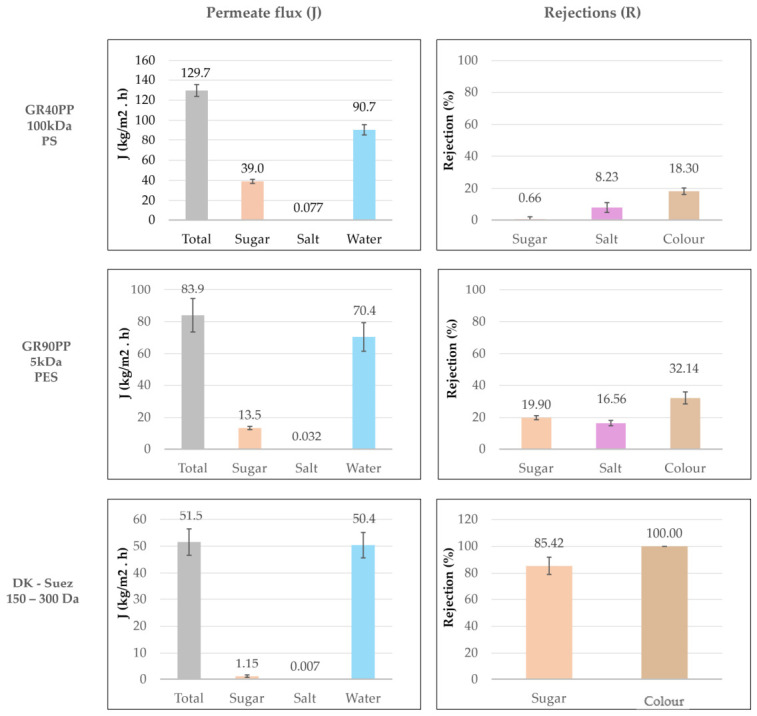
Confirmatory performance of selected membranes under optimal conditions. Total and specific permeate fluxes (sugar, salt, water) and rejection coefficients are shown for UF (GR40PP, 100 kDa; GR90PP, 5 kDa) and NF (DK-Suez, 150–300 Da) membranes. Values represent mean ± standard deviation (*n* = 5); no significant differences were observed (*p* > 0.05).

Among the NF membranes, DK-Suez was selected as the most suitable membrane. Although its separation performance was broadly comparable to that of the Alfa Laval membrane, it showed markedly better fouling resistance and is designed for operation at temperatures up to 80 °C, which is advantageous for integration into starch hydrolysate processing. Furthermore, Dk-Suez provided high sugar retention (85.4%), making it particularly suitable for product concentration and the removal of low-molecular-weight impurities while preserving the desired carbohydrate profile. By contrast, DL-Suez was excluded despite its meaningful colour permeation because this behaviour was accompanied by considerable sugar losses, indicating limited sugar/colour selectivity, which makes it unsuitable for the intended application where the retentate constitutes the final product stream. The reproducibility of the selected operating conditions was verified through replicate experiments, the results of which are presented in Figure 8. Under the selected conditions, the final permeate fluxes were 129.7, 83.9, and 51.5 kg·m^−2^·h^−1^ for the loose UF (GR40PP, 100 kDa), tight UF (GR90PP, 5 kDa), and NF (DK-Suez, 150–300 Da) membranes, respectively. The loose and tight UF steps provided partial decolourisation, with colour removals of 18.3% and 32.0%, respectively. The NF stage did not contribute substantially to colour removal; however, it effectively concentrated the product, achieving a sugar rejection of 85.4% and facilitating the removal of low molecular-weight impurities. Statistical analysis (ANOVA) showed no significant differences among replicates (*p* > 0.05), demonstrating the robustness and reproducibility of the selected membrane configuration.

The choice of elevated temperature and pressure for the UF stages was supported not only by the screening results but also by broader process considerations. Higher temperatures reduce viscosity, enhance mass transfer, and support hygienic operation by limiting microbial growth, while higher pressures ensure sufficient driving force in sugar-rich systems. These conditions are therefore particularly attractive for industrial implementation, provided that membrane stability and fouling remain under control.

### 3.4. Product Quality and Carbohydrate Fractionation

**Figure 9:**
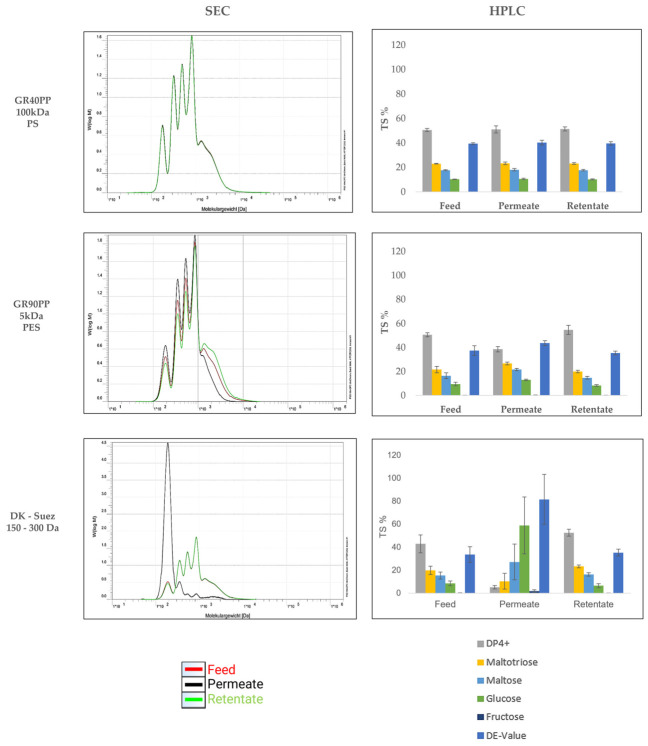
SEC and HPLC analysis of carbohydrate composition before and after membrane filtration. (**Left**): SEC profiles of feed, permeate, and retentate showing molecular weight distribution. (**Right**): HPLC quantification of sugar fractions (DP4+, maltotriose, maltose, glucose, fructose) and corresponding DE values. Results illustrate membrane-induced changes in composition and confirm compliance with DE40 specifications.

To verify that membrane treatment did not adversely affect product quality, the selected membranes were further evaluated using size exclusion chromatography (SEC) and detailed carbohydrate profiling by HPLC. The combined results are shown in Figure 9.

For the loose UF membrane, GR40PP (100 kDa), the carbohydrate profiles of feed, permeate, and retentate were nearly identical, indicating that no measurable fractionation of the carbohydrate spectrum occurred. This behaviour was expected given the large pore size of the membrane, which allowed essentially all carbohydrate fractions to pass into the permeate.

The tight UF membrane, GR90PP (5 kDa), showed limited fractionation. Although small differences in molecular-weight distribution were detected, the overall carbohydrate profile remained within the specification range for DE40 syrup. The most noticeable changes occurred in the intermediate molecular-weight region (approximately 10^4^–10^5^ Da), which may correspond to the partial removal of residual proteins and colour-forming compounds such as melanoidins rather than to major sugar fractionation [49]. Although protein and amino acid concentrations were not quantified in the present study, residual proteins are expected to be present in low concentrations following the upstream clarification process and may contribute to membrane retention and flux reduction phenomena through adsorption or gel-layer formation. This supports the interpretation that the membrane achieved impurity removal without substantially altering the desired sugar composition.

The NF membrane, DK-Suez, induced a greater degree of fractionation, as expected from its tighter separation range. Lower-molecular-weight sugars such as glucose and maltose preferentially permeated the membrane, while higher-molecular-weight carbohydrates were enriched in the retentate. Nevertheless, the retentate remained within DE40 product specifications, indicating that NF improved concentration and impurity removal without compromising overall product quality.

Taken together, the SEC and HPLC analyses confirm that the selected membrane sequence—loose UF, tight UF, followed by NF—can improve starch hydrolysate quality while preserving the essential carbohydrate composition of the product. These compositional results complement the bulk performance indicators and demonstrate that gains in decolourisation or purification were not achieved at the expense of unacceptable changes in sugar profile.

## 4. Conclusions

This study systematically evaluated polymeric ultrafiltration and nanofiltration membranes for the decolourisation and purification of starch hydrolysates and established a suitable membrane sequence for further process development. Among the membranes screened, the most promising configuration consisted of a loose UF membrane (GR40PP, 100 kDa) for initial clarification, followed by a tight UF membrane (GR90PP, 5 kDa) for enhanced decolourisation, and a tight NF membrane (DK-Suez, 150–300 Da) as complementary concentration, and conditioning step, providing high sugar retention and enabling further processing of carbohydrate-rich retentate stream. The most favourable operating conditions were identified as 60 °C and 8 bar for UF, and 30 bar, 60 °C, and 15 °Brix for NF.

The results showed that UF was the most relevant step for colour reduction, achieving partial decolourisation of approximately 18% with the loose UF membrane and 32% with the tight UF membrane, while maintaining high permeate fluxes and acceptable sugar losses. In contrast, NF contributed only limited additional decolourisation but proved effective for sugar retention, product concentration, and further purification. The evaluated NF membranes generally exhibited similar sugar and colour rejection behaviour, indicating limited sugar/colour selectivity and therefore limited suitability as a stand-alone decolourisation technology. Instead, NF was found to be more appropriately applied as a complementary polishing and concentration step following UF treatment. Overall, the findings confirmed that no single membrane can simultaneously maximise colour removal, sugar recovery, and impurity control, highlighting the importance of combining complementary membrane stages.

Replicate experiments confirmed the reproducibility and stability of the selected membranes under the chosen operating conditions, with no significant differences between runs (*n* = 5, *p* > 0.05). In addition, the selected membranes exhibited low flux reduction tendencies and sufficient thermal robustness under the investigated operating conditions, supporting their suitability for longer-term operation and potential industrial integration.

These results provide a robust basis for the next stage of research, in which the selected membrane configuration can be further validated and integrated into a multistep purification strategy for improved starch hydrolysate processing. Although cleaning strategies were not evaluated within the scope of the present screening study, subsequent long-term investigations identified organic fouling as the predominant performance-limiting mechanism and demonstrated that alkaline cleaning was the most effective approach for membrane performance recovery [50]. Future research should therefore focus on optimising cleaning protocols and evaluating alternative fouling-mitigation strategies, including enzymatic treatments and milder chemical formulations, to further improve membrane longevity, process sustainability, and industrial applicability.

## Figures and Tables

**Table 1:** Physicochemical characterisation of DE40 starch hydrolysate syrup.

Parameters	Value
Dry matter (%)	72
Protein Kjeldahl (% i. TS)	0.12
Conductivity (µS/cm)	633
pH	5.73
Colour (IU)	742
Ion Analysis (% i. TS)	
Chloride	0.03
Nitrate	<0.01
Citrate	
Sulphate	0.11
Phosphate	0.016
Sodium	0.090
Calcium	0.004
Magnesium	0.004
Carbohydrate spectrum (% i. TS)	
DP4+	46.92
Maltotriose	22.32
Maltose	19.10
Glucose	9.55
5-Hydroxymethylfurfural (mg/kg)	16.94

**Table 2:** Main Characteristics of the UF and NF membranes tested.

Characteristic	GR80PP	GR90PP	GR40PP	GR61PP	HFM140	NF	DL	DK
Manufacturer	Alfa Laval				Koch	Alfa Laval	Suez	
Material	Polyethersulphone PES		Polysulphone PSU		PVDF	Thin film membranes		
MWCO	10 kDa	5 kDa	100 kDa	20 kDa	70 kDa	150–300		
Operating temperature (°C)	5–75				5–60	60	80	
Operating pH range	1–13				2–10	3–10	2–10	
Maximum pressure (bar)	1–10				2–8	55	40	

**Table 3:** Operating conditions for the single screening test.

**Ultrafiltration**	
Temperature (°C)	40 and 60
Pressure (bar)	2 and 8
Feed Concentration (°Brix)	20 and 30
**Nanofiltration**	
Temperature (°C)	40 and 60
Pressure (bar)	30
Feed Concentration (°Brix)	15 and 20

**Table 4:** Comparative summary of UF and NF membrane screening results and final membrane selection.

Process	Membrane	Flux (kg·m^−2^·h^−1^)	Colour Removal (%)	Sugar Loss (%)	Further Purification (%)	Flux Reduction (%)	Selection Final Score
Tight UF	GR90PP (5 kDa)	Moderate	High	Moderate	Moderate	Moderate	✔ = 16
	GR80PP (10 kDa)	Low	Moderate	Moderate	Very Low	Moderate	✘ = 12
	GR61PP (20 kDa)	Low	Moderate	Moderate	Very Low	Moderate	✘ = 12
Loose UF	HFM140 (70 kDa)	Moderate	Moderate	Low	Low	Low	✘ = 16
	GR40PP (100 kDa)	High	Moderate	Very Low	Low	Moderate	✔ = 17
NF	Alfa Laval (<300 Da)	Moderate	Very Low	Very Low	High	Low	✘ =17
	DL Suez (150–300 Da)	High	Moderate	High	Moderate	Moderate	✘ = 12
	DK Suez (150–300 Da)	Moderate	Very Low	Very Low	High	Very Low	✔ = 18

Qualitative performance indicators were converted into numerical scores using a semi-quantitative KPI approach; only the final overall score is reported in the table for clarity. Flux reduction (%) was calculated from the difference between the pure water flux measured before and after filtration and is used as a qualitative indicator of apparent fouling tendency and membrane performance loss. Green highlighting (✓) indicates the membrane(s) selected for the subsequent process based on the overall performance score. Red highlighting (✗) indicates membranes that were not selected.

## Data Availability

Data is unavailable due to privacy or ethical restrictions.

## References

[1] Shahidi Noghabi M., Razavi S.M.A. (2015). Increase the Quality of Sugar by Ultrafiltration Process. J. Food Process. Preserv..

[2] Wang H.-Y., Qian H., Yao W.-R. (2011). Melanoidins Produced by the Maillard Reaction: Structure and Biological Activity. Food Chem..

[3] Bhattacharya P.K., Agarwal S., De S., Rama Gopal U.V.S. (2001). Ultrafiltration of Sugar Cane Juice for Recovery of Sugar: Analysis of Flux and Retention. Sep. Purif. Technol..

[4] Hobbs L. (2009). Sweeteners from Starch: Production, Properties and Uses. Starch.

[5] Gyura J., Šereš Z., Vatai G., Molnár E.B. (2002). Separation of Non-Sucrose Compounds from the Syrup of Sugar-Beet Processing by Ultra- and Nanofiltration Using Polymer Membranes. Desalination.

[6] Cassano A., Drioli E. (2014). Integrated Membrane Operations in the Food Production.

[7] Guo S., Luo J., Yang Q., Qiang X., Feng S., Wan Y. (2019). Decoloration of Molasses by Ultrafiltration and Nanofiltration: Unraveling the Mechanisms of High Sucrose Retention. Food Bioprocess Technol..

[8] Cabeza C.A., El-Gohary-Ahmed A., Minauf M., Harasek M. (2022). Sustainable Industrial Treatment of Starch Hydrolysates. Chem. Eng. Trans..

[9] Guo S., Luo J., Wu Y., Qi B., Chen X., Wan Y. (2018). Decoloration of Sugarcane Molasses by Tight Ultrafiltration: Filtration Behavior and Fouling Control. Sep. Purif. Technol..

[10] Qiang X., Luo J., Guo S., Cao W., Hang X., Liu J., Wan Y. (2019). A Novel Process for Molasses Utilization by Membrane Filtration and Resin Adsorption. J. Clean. Prod..

[11] Hamachi M., Gupta B.B., Ben Aim R. (2003). Ultrafiltration: A Means for Decolorization of Cane Sugar Solution. Sep. Purif. Technol..

[12] Qi B., Wu Y., Guo S., Luo J., Wan Y. (2017). Refinement of Cane Molasses with Membrane Technology for Clarification and Color Removal. J. Membr. Sci. Res..

[13] Commission E. Evaluation of EU Legislation on Urban Waste Water Treatment Finds That It Is Fit for Purpose but Its Effectiveness Could Be Improved. https://eur-lex.europa.eu/legal-content/EN/TXT/?uri=CELEX%3A52019SC0700&qid=1690378833708.

[14] Gyura J., Šereš Z., Eszterle M. (2005). Influence of Operating Parameters on Separation of Green Syrup Colored Matter from Sugar Beet by Ultra-and Nanofiltration. J. Food Eng..

[15] Djurić M., Gyura J., Zavargo Z. (2004). The Analysis of Process Variables Influencing Some Characteristics of Permeate from Ultra- and Nanofiltration in Sugar Beet Processing. Desalination.

[16] LeBlanc J., Vu T., Aguda R. (2026). Review of the Technical Readiness of Ultrafiltration Membrane-Based Sugarcane Juice Clarification and Subsequent Sugar Production. J. Food Eng..

[17] Luo J., Hang X., Zhai W., Qi B., Song W., Chen X., Wan Y. (2016). Refining Sugarcane Juice by an Integrated Membrane Process: Filtration Behavior of Polymeric Membrane at High Temperature. J. Memb. Sci..

[18] Goulas A.K., Kapasakalidis P.G., Sinclair H.R., Rastall R.A., Grandison A.S. (2002). Purification of Oligosaccharides by Nanofiltration. J. Memb. Sci..

[19] Luo J., Wan Y. (2013). Effects of PH and Salt on Nanofiltration—A Critical Review. J. Memb. Sci..

[20] Hinkova A., Bohacenko I., Bubnik Z., Hrstkova M., Jankovska P. (2004). Mineral Membrane Filtration in Refinement of Starch Hydrolysates. J. Food Eng..

[21] Gotsmy R. (2021). Decolourisation of Starch Hydrolysates Using Ultrafiltration. Master’s Thesis.

[22] Cabeza C.A., Ahmed A.E.G., Minauf M., Harasek M. (2022). Integration of Membrane Processes for Decolourization of Starch Hydrolysates. Chem. Eng. Trans..

[23] Hoàng V., Duc T. (2022). Response Surface Optimization of Enzymatic Hydrolysis of Germinated Brown Rice for Higher Reducing Sugar Production. Vietnam J. Food Control.

[24] Acevedo-Estupiñan M.V., Parra-Escudero C.O., Muvdi-Nova C.J. (2015). Study of Clarification Process of Cassava Starch Hydrolysates Using Ceramic Membranes. Vitae.

[25] Shahabi-Ghahfarrokhi I., Goudarzi V., Babaei-Ghazvini A. (2019). Production of Starch Based Biopolymer by Green Photochemical Reaction at Different UV Region as a Food Packaging Material: Physicochemical Characterization. Int. J. Biol. Macromol..

[26] Sivamaruthi B.S., Nallasamy P.K., Suganthy N., Kesika P., Chaiyasut C. (2022). Pharmaceutical and Biomedical Applications of Starch-Based Drug Delivery System: A Review. J. Drug Deliv. Sci. Technol..

[27] Widiasa I., Wenten I.G. (2005). Fouling Behaviour During Cross Flow Ultrafiltration of Cassava Starch Hydrolysate Using Polyacrilonitrile Membrane. J. Appl. Membr. Sci. Technol..

[28] Cabeza C., Ahmed A.E.G., Minauf M., Wieland K., Harasek M. (2025). Starch Hydrolysates, Their Impurities and the Role of Membrane-Based Technologies as a Promising Sustainable Purification Method at Industrial Scale. Food Res. Int..

[29] Giani S. (2018). Determination of Sugar Solutions Color According to ICUMSA/Application Note Analytical Chemistry.

[30] Elewa M., El-Saady G., Ibrahim K., Tawfek M., Elhossieny H. (2020). A Novel Method for Brix Measuring in Raw Sugar Solution. Egypt. Sugar J..

[31] Martí-Calatayud M.-C., Vincent-Vela M.-C., Álvarez-Blanco S., Lora-García J., Bergantiños-Rodríguez E. (2010). Analysis and Optimization of the Influence of Operating Conditions in the Ultrafiltration of Macromolecules Using a Response Surface Methodological Approach. Chem. Eng. J..

[32] Alventosa-De Lara E., Barredo-Damas S., Alcaina-Miranda M.I., Iborra-Clar M.I. (2012). Evolution of Membrane Performance during the Ultrafiltration of Reactive Black 5 Solutions: Effect of Feed Characteristics and Operating Pressure. Chem. Eng. Trans..

[33] Cheryan M. (1998). Ultrafiltration and Microfiltration Handbook.

[34] Leo C.P., Cathie Lee W.P., Ahmad A.L., Mohammad A.W. (2012). Polysulfone Membranes Blended with ZnO Nanoparticles for Reducing Fouling by Oleic Acid. Sep. Purif. Technol..

[35] Fathanah U., Rosnelly C.M., Zuhra Z., Muchtar S., Rinaldi W., Abubakar A., Rahmah F., Ambarita A.C., Yusuf M., Ramadhani D.S. (2025). Synthesis and Characterization of Hydrophobic Polyethersulfone Membranes Modified by Hydrophilic Additives by NIPS Method. IOP Conf. Ser. Earth Environ. Sci..

[36] Liu F., Hashim N.A., Liu Y., Abed M.R.M., Li K. (2011). Progress in the Production and Modification of PVDF Membranes. J. Memb. Sci..

[37] Zhu Z., Luo X., Yin F., Li S., He J. (2018). Clarification of Jerusalem Artichoke Extract Using Ultra-Filtration: Effect of Membrane Pore Size and Operation Conditions. Food Bioprocess Technol..

[38] Tsuru T., Izumi S., Yoshioka T., Asaeda M. (2000). Temperature Effect on Transport Performance by Inorganic Nanofiltration Membranes. AIChE J..

[39] Tong X., Zhao X.-H., Wu Y.-H., Bai Y., Ikuno N., Ishii K., Hu H.-Y. (2021). The Molecular Structures of Polysaccharides Affect Their Reverse Osmosis Membrane Fouling Behaviors. J. Memb. Sci..

[40] Freger V., Arnot T.C., Howell J.A. (2000). Separation of Concentrated Organic/Inorganic Salt Mixtures by Nanofiltration. J. Memb. Sci..

[41] Wang X.-L., Zhang C., Ouyang P. (2002). The Possibility of Separating Saccharides from a NaCl Solution by Using Nanofiltration in Diafiltration Mode. J. Memb. Sci..

[42] Huang J.-H., Shi L.-J., Zeng G.-M., Li X., He S.-B., Li F., Xiong Y.-L., Guo S.-H., Zhang D.-M., Xie G.-X. (2012). Effects of Feed Concentration and Transmembrane Pressure on Membrane Fouling in Cd2+ Removal by Micellar-Enhanced Ultrafiltration. Desalination.

[43] Sutzkover I., Hasson D., Semiat R. (2000). Simple Technique for Measuring the Concentration Polarization Level in a Reverse Osmosis System. Desalination.

[44] Samee M.A., Elgohary A.A., Harasek M., Friedl A. (2016). Experimental Investigation of Nanofiltration Process for the Separation of Complex Sugar Mixtures Containing Mono- and Multivalent Salts. Chem. Eng. Trans..

[45] Luo J., Guo S., Qiang X., Hang X., Chen X., Wan Y. (2019). Sustainable Utilization of Cane Molasses by an Integrated Separation Process: Interplay between Adsorption and Nanofiltration. Sep. Purif. Technol..

[46] Field R.W., Wu D., Howell J.A., Gupta B.B. (1995). Critical Flux Concept for Microfiltration Fouling. J. Memb. Sci..

[47] Bacchin P., Aimar P., Field R.W. (2006). Critical and Sustainable Fluxes: Theory, Experiments and Applications. J. Memb. Sci..

[48] Van der Bruggen B., Curcio E., Drioli E. (2004). Process Intensification in the Textile Industry: The Role of Membrane Technology. J. Environ. Manag..

[49] Ahmed A.E., Jordan C., Walcher E., Kuloglija S., Turetschek R., Lozar A., Tomasetig D., Harasek M. (2025). Membrane Processes for Remediating Water from Sugar Production By-Product Stream. Membranes.

[50] Cabeza C., Ahmed A.E.G., Minauf M., Wieland K., Harasek M. (2025). Enhancing Starch Hydrolysate Syrup Purification: Long-Term Ultrafiltration Membrane Performance Under Industrial Conditions. Sep. Purif. Technol..

